# Translucency and colour masking ability of hybrid CAD/CAM materials with different thicknesses: in vitro study

**DOI:** 10.1038/s41405-024-00245-0

**Published:** 2024-08-05

**Authors:** Zeinab Omar Tolba, Eman Ezzat Youssef Hassanien

**Affiliations:** 1 Lecturer, Conservative Dentistry Department, Faculty of Dentistry, Cairo University, Cairo, Egypt; 2 Lecturer, Fixed Prosthodontics Department, Faculty of Dentistry, Cairo University, Cairo, Egypt

**Keywords:** Health care, Dentistry

## Abstract

**Objective:**

To evaluate effect of material thickness and translucency of two different hybrid CAD/CAM materials on their masking ability.

**Materials and Methods:**

40 rectangular-shaped specimens (12 × 14 mm) were obtained from Vita Enamic and Grandio blocks at 0.5 and 1 mm-thick (n = 10/group). Colour parameters of specimens were measured on PMMA tooth-coloured replica backgrounds (A2, C4) and black, white backings using dental spectrophotometer. Translucency parameter (TP) and masking ability (∆E_00_) values of tested materials with 0.5, and 1 mm thicknesses were calculated. Quantitative variables were compared between groups using student t-test.

**Results:**

TP and ∆E_00_ significantly decreased as thickness of tested materials increased. Grandio showed significantly higher TP values. There were significantly lower ΔE_00_ values for Vita Enamic compared to Grandio at 1 mm thickness and inversely insignificant at 0.5 mm. The 0.5 mm-thick ∆E_00_ of tested materials was above clinical acceptability threshold, whereas, 1 mm-thick did not exceed showing better masking properties.

**Conclusions:**

Thickness is more effective for colour masking than translucency. In thin thickness, the masking ability is less effective, irrespective of tested materials. Translucency of tested materials was affected by their composition. Both hybrid CAD/CAM materials are promising alternatives for masking dark discolouration at 1 mm-thick.

## Introduction

Patients’ demand for aesthetic dentistry has recently been raised as a first requisite, particularly laminate veneers, which positively impact their image and self-esteem [1, 2]. The improper restoration colour match to the adjacent natural teeth, especially in cases of discoloured teeth, can jeopardise aesthetic success [3, 4]. Restorations with higher masking abilities and less translucency are required in many case scenarios [5]. Additionally, practitioners have to consider which kind of aesthetic material effectively masks and recovers the optical properties of natural teeth [6]. Several studies have shown a strong correlation between the masking effect and the thickness of the restoration [7–10]. Minimally invasive cosmetic dentistry (MICD) and minimally invasive prosthetic procedure (MIPP) are well-known aesthetic dentistry concepts that prioritise maximum tooth tissue preservation [11]. Hybrid materials, combining all ceramic and composite properties, are being introduced to CAD/CAM-based restorative dentistry, enhancing optical and mechanical properties for indirect restoration fabrication [12, 13]. Studies have revealed their superb fatigue resistance, enabling the fabrication of ultra-thin, non-invasive restorations, in addition to enhanced machinability and intra-oral repair capabilities [3, 13].

The characteristic properties of resin-matrix ceramic (RMC) materials are influenced by factors like microstructure, filler particle volume and size, resin-matrix content and manufacturing technique. Consistently, as industrial technologies progress, new chemical formulations are continuously being developed for RMCs [14]. RMCs can be categorised as resin nanoceramic or polymer-infiltrated ceramic-network materials based on the way ceramics are incorporated into the polymeric matrix [3]. Vita Enamic (VITA Zahnfabrik, Bad Sackingen, Germany) is the first hybrid ceramic material, consisting of a sintered ceramic matrix (86% in weight) infiltrated with a polymer matrix (14% in weight). Enamic mimics natural dentine properties such as wear, flexural and elasticity, in addition to being strong in thin sections as well as having variable translucency [5, 15]. While, Grandio (VOCO GmbH, Cuxhaven, Germany) is a highly filled (86%) CAD/CAM restorative material that is based on nanoceramic hybrid technology [16].

Optimal translucency is required to achieve lifelike restorations and is therefore crucial during the selection of materials [17]. The translucency parameter (TP) value can be influenced by factors such as filler particle size, content and amount, material thickness, surface texture, metal oxides and underlying foundation [14]. All these variables can alter light transmittance due to absorption and scattering of the incident light, resulting in colourimetric differences [3].

The types of resin-matrix ceramics and the colour of the underlying structures can alter the final optical features of indirect restorations in a clinical setting [3]. The literature provides diverse information on the translucency and final colour of predecessor RMCs, but data on newly introduced RMCs is scant to the authors’ knowledge [14]. Moreover, some authors reveal that masking a dental substrate with cements may not be feasible due to lack of different shades, and thin cement layers [6]. Thus, the aim of this study was to evaluate the effect of material thickness and translucency of two different hybrid CAD/CAM materials on their masking ability, with the null hypothesis suggesting no difference between the two materials.

## Materials and methods

### Ethical approval and preparation of specimens

The Faculty of Dentistry Research Ethics Committee (Committee 10/2022) at Cairo University granted ethical approval for this in vitro study. Two types of hybrid CAD/CAM blocks (Vita Enamic and Grandio) were used in this study, Table 1. The blocks were sliced into rectangular-shaped plates (12 × 14 mm) with 0.5 and 1 mm thicknesses (n = 10 for each group) using a double-sided Isomet 4000 micro-saw (Buehler, USA) under constant water cooling, with a total of 40 samples. Polishing of the specimens was performed using two-steps polishing system with pre- and high-gloss polishers (Vita Enamic Polishing set, VITA Zahnfabrik, Bad Sackingen, Germany). The final thicknesses of the specimens were measured by a digital caliper (INSIZE, China). Prior to colour measurements, all specimens were ultrasonically cleaned in distilled water for 10 min (CODYSON ultrasonic cleaner, CD-4820, China) and dried with compressed air. The sample size for this study was determined based on data obtained from a previous study [18] and calculated by using a statistical software (PS programme version 3.1.2), power of 80% and a 5% alpha level of significance and an effect size of 1.3249474. The calculated sample size was 40 specimens (10 per group).

**Table 1:** Materials, description, composition, manufacturer and shade of tested materials.

Material	Description	Composition	Manufacturer	Shade
Vita Enamic	PICN (polymer-infiltrated ceramic network). Hybrid ceramic with a dual network structure that combines the best characteristics of ceramic and composite.	The dominant fine-structure ceramic network (~86% by weight) is reinforced by an acrylate polymer network (~14% by weight, UDMA + TEGDMA), with both networks penetrating completely.	VITA Zahnfabrik, Bad Sackingen, Germany	T 2M2
Grandio blocs	Nanoceramic hybrid composite CAD/CAM block.	86% (by weight) Nanohybrid fillers, 14% UDMA + DMA	VOCO GmbH, Cuxhaven, Germany	LT A2

### Underlying background

Four backgrounds were used in the study: black and white, and two different backgrounds simulated two conditions, a severely discoloured (C4) and a tooth surface with no discolouration (A2). The CIELAB coordinates (L* a* b*) of black (L = 25.96, a = 0.67, b = −0.80) and white (L = 92.75, a = 1.97, b = −9.10) cards were obtained using a spectrophotometer (UltraScan PRO, HunterLab, Reston, USA). The tooth-coloured backgrounds (rectangular-shaped, 12 × 14 × 10 mm-thick) were sliced from PMMA disc shade A2 (On-dent, Izmir, Turkey) using a double-sided Isomet 4000 micro-saw under constant water cooling. The discoloured background (C4) was obtained by staining the rectangular-shape A2 using the OPTIGLAZE colour kit (GC, Japan) according to manufacturer instructions and verified by the spectrophotometer (VITA Easyshade V, Zahnfabrik, Bad Sackingen, Germany). The staining was performed as follows: sandblasting of the surface (Basic Eco Fine Sandblasting Unit, Renfert GmbH, Germany) with 50 μm Al_2_O_3_ particles (1.5 bar), cleaning, drying, followed by the application of stain in two coats, each cured for 90 s using bre.Lux Power Unit 2 (bredent, GmbH & Co. KG., Senden, Germany).

### Colour measurement of hybrid CAD/CAM specimens with different thicknesses

The shade of different thicknesses of both hybrid CAD/CAM materials against the four backgrounds without an underlying medium were measured by the spectrophotometer (VITA Easyshade V) in ‘tooth single’ mode. The colour parameters were determined in three coordinate dimensions: L* (from 0 [black] to 100 [white]), a* red–green (+a* = red; −a* = green) and b* yellow–blue (+b* = yellow; −b* = blue). All measurements were performed by the same operator and under the same illumination, to exclude any variation. The illumination condition in the dental clinic was set at standardised daylight with a colour temperature of 6500 K (PHILIPS Corp.) and natural direct daylight was excluded. During all measurements, the spectrophotometer measuring tip was positioned perpendicular to the centre of each specimen with full contact to ensure standardisation. The Easyshade V was recalibrated with its in-built apparatus prior to each specimen measurement. Measurements were repeated three times for each specimen on each background and the mean CIE L*a*b* values were recorded.

### Translucency parameter measurement

Colour coordinates of each sample were independently measured on white and black backgrounds. The translucency parameter (TP) of materials was calculated with the following equation: ${{{\rm{TP}}}}=\sqrt{{\left({{{{\rm{L}}}}}_{{{{\rm{b}}}}}-{{{{\rm{L}}}}}_{{{{\rm{w}}}}}\right)}^{2}+{\left({{{{\rm{a}}}}}_{{{{\rm{b}}}}}-{{{{\rm{a}}}}}_{{{{\rm{w}}}}}\right)}^{2}+{\left({{{{\rm{b}}}}}_{{{{\rm{b}}}}}-{{{{\rm{b}}}}}_{{{{\rm{w}}}}}\right)}^{2}}$, where b and w represent colour coordinates against black and white backgrounds, respectively, with TP values ranging from 0 to 100, indicating its opaqueness or transparency. The greater the TP value, the higher the translucency of the material [19].

### Masking ability measurement

The same specimen was placed over the discoloured C4 (L = 59.4, a = 1.2, b = 22.3) and A2 (L = 77.2, a = −0.3, b = 20.8) backgrounds. The colour difference ∆E_00_ of each specimen on a discoloured background in relation to the background A2 was calculated with CIEDE2000 (2:1:1) formula [20, 21] as follows:

$${\triangle {{{\rm{E}}}}}_{00}=\sqrt{{\left(\frac{\triangle {{{{\rm{L}}}}}^{{\prime} }}{{{{{\rm{K}}}}}_{{{{\rm{L}}}}}{{{{\rm{S}}}}}_{{{{\rm{L}}}}}}\right)}^{2}+{\left(\frac{\triangle {{{{\rm{C}}}}}^{{\prime} }}{{{{{\rm{K}}}}}_{{{{\rm{C}}}}}{{{{\rm{S}}}}}_{{{{\rm{C}}}}}}\right)}^{2}+{\left(\frac{\triangle {{{{\rm{H}}}}}^{{\prime} }}{{{{{\rm{K}}}}}_{{{{\rm{H}}}}}{{{{\rm{S}}}}}_{{{{\rm{H}}}}}}\right)}^{2}+{{{{\rm{R}}}}}_{{{{\rm{T}}}}}\left(\frac{\triangle {{{{\rm{C}}}}}^{{\prime} }}{{{{{\rm{K}}}}}_{{{{\rm{C}}}}}{{{{\rm{S}}}}}_{{{{\rm{C}}}}}}\right)\left(\frac{\triangle {{{{\rm{H}}}}}^{{\prime} }}{{{{{\rm{K}}}}}_{{{{\rm{H}}}}}{{{{\rm{S}}}}}_{{{{\rm{H}}}}}}\right)}$$

where ∆L’, ∆C’, ∆H’: metric differences between the corresponding values of the samples, based on uniform colour space used in CIEDE2000. R_T_: rotation function that explains the interaction between the differences of chroma and hue in the blue region. K_L_S_L_, K_C_S_C_ and K_H_S_H_: empirical terms used to correct (weight) the metric differences to the CIEDE2000 differences for each coordinate. Parametric factors were set to K_L_ = 2, K_H_ = 1 and K_C_ = 1 for CIEDE2000 (2:1:1). Colour difference values were compared only with the 50:50% acceptability thresholds (AT) for CIEDE2000 (2:1:1) as 1.78 units [20–23].

In the present study, colour change values were calculated with the CIEDE2000 (2:1:1) formula, as advised by Paravina et al. [22, 23] and Perez et al. [20]. The CIEDE2000 formula performed better using K_L_, K_C_ and K_H_ parametric factors set to 2:1:1 than 1:1:1, which recommends its usage in dental research and in vivo instrumental colour analysis [20].

In most of the studies evaluating the optical properties of materials, researchers preferred the use of materials of A2 colour [24–26], so A2 was used in the current study to simulate tooth colour. We also used a C4-shade background to simulate a severely discoloured tooth and evaluated the ability of different thicknesses of Grandio versus Vita Enamic specimens to mask this discolouration by measuring their CIEDE2000 values.

### Statistical analysis

Data was entered and statistically analysed on the Statistical Package of Social Science Software programme, version 25 (IBM SPSS Statistics for Windows, Version 25.0. Armonk, NY: IBM Corp.). Data was presented using mean and standard deviation for quantitative variables. Comparison between groups for quantitative variables was performed using student t-test. The Effect size was performed using the mean difference and its corresponding 95% confidence interval. P values less than or equal to 0.05 were considered statistically significant.

## Results

### Translucency parameter

**Fig. 1:**
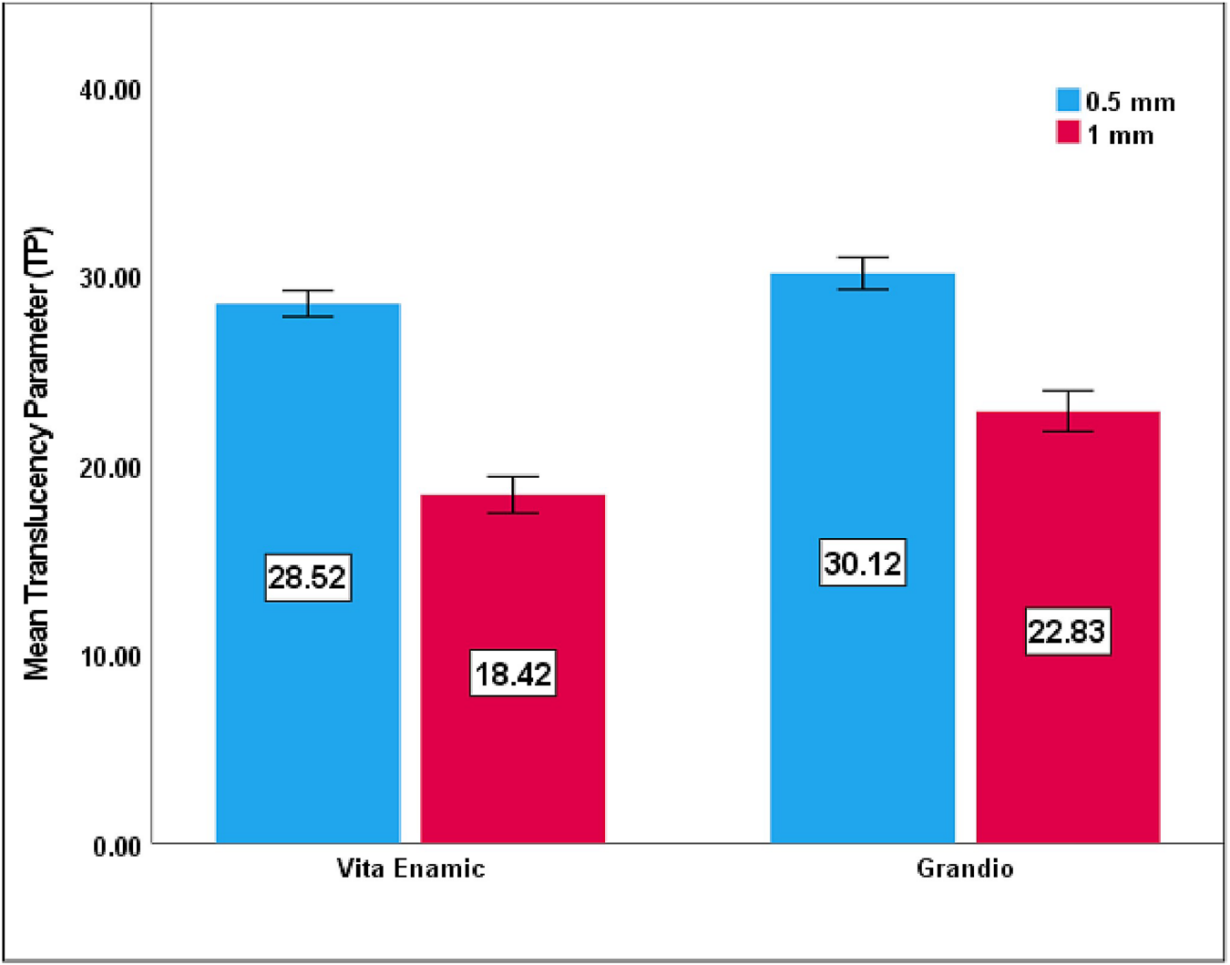
Mean TP value of Vita Enamic and Grandio at 0.5 and 1 mm-thick.

Comparisons between Vita Enamic and Grandio and within each material at different thicknesses were outlined in Table 2, Fig. 1. Increasing material thickness significantly decreased TP value for each hybrid CAD/CAM tested material (p < 0.001 for each). Regarding the 0.5 and 1 mm thicknesses, Grandio showed statistically significant higher TP values (30.12 ± 0.42, 22.83 ± 0.54, respectively) compared to Vita Enamic (28.52 ± 0.35, 18.42 ± 0.48, respectively), with p < 0.001 for each thickness separately.

**Table 2:** Comparison of Translucency between Vita Enamic and Grandio at 0.5 mm, 1 mm thicknesses separately and different thicknesses within each material

Materials	Thickness		Mean difference (95% CI)	P value
	0.5 mm	1 mm		
Vita Enamic	28.52 ± 0.35	18.42 ± 0.48	10.10 (9.71–10.49)	<0.001
Grandio	30.12 ± 0.42	22.83 ± 0.54	7.29 (6.84–7.75)	<0.001
Mean difference (95% CI)	1.60 (1.24–1.97)	4.41 (3.93–4.89)		
P value	<0.001	<0.001

### Colour difference

**Fig. 2:**
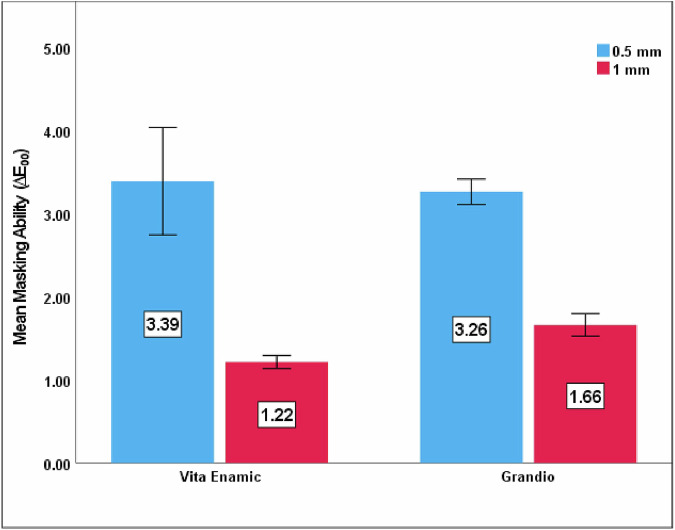
Mean ∆E_00_ value (masking ability) of Vita Enamic and Grandio at 0.5 and 1 mm-thick.

Table 3 and Fig. 2 show the comparisons of tested materials regarding masking ability. Same as translucency parameter, ΔE_00_ values showed a decrease with an increase in material thickness, with significant difference observed (p < 0.001 for each). At 1 mm-thick, ΔE_00_ values for Vita Enamic (1.22 ± 0.04) were significantly lower than Grandio (1.66 ± 0.07), with p < 0.001. Meanwhile, at 0.5 mm-thick, Grandio (3.26 ± 0.08) showed lower ΔE_00_ than Vita Enamic (3.39 ± 0.32), yet with no significant difference (p = 0.243). The tested materials did not exceed the clinical acceptability threshold for 1 mm-thick specimens, but the 0.5 mm-thick specimens exceeded it.

**Table 3:** Comparison of Masking ability between Vita Enamic and Grandio at 0.5 mm, 1 mm thicknesses separately and different thicknesses within each material

Materials	Thickness		Mean difference (95% CI)	P value
	0.5 mm	1 mm		
Vita Enamic	3.39 ± 0.32	1.22 ± 0.04	2.18 (1.96–2.39)	<0.001
Grandio	3.26 ± 0.08	1.66 ± 0.07	1.60 (1.53–1.67)	<0.001
Mean difference (95% CI)	−0.13 (−0.35–0.9)	0.44 (0.02–0.39)		
P value	0.243	<0.001

## Discussion

Colour and translucency parameters strongly influence the aesthetic outcome of dental restorations [27]. The primary factor to control colour is the underlying dark tooth structure, which can result in the darkening of restoration [28, 29]. Moreover, maintaining a proper balance between translucency and restoration thickness is crucial to mimic natural teeth’s appearance [27, 30, 31]. Practitioners should keep in mind the clue of minimally invasive tooth preparation [11]. Moreover, there is limited information about the optical properties of hybrid CAD/CAM materials in the literature [19]. Therefore, the current study was conducted to evaluate the effect of material thickness and translucency of two different hybrid CAD/CAM materials (Vita Enamic and Grandio) on their masking ability.

It was mentioned in previous studies that the main components that significantly influence translucency of the restoration are thickness of material [19, 32], filler particle size [33], the type of metal oxides [33, 34] surface texture [35] and the characteristics of the underlying foundation [36].

In the present study, both materials revealed a higher significant mean TP for 0.5 mm than a 1 mm-thick specimen. This may be attributed to a decrease in material thickness, which permits more light to be transmitted through the thinner thickness with less attenuation [19, 24, 32]. Barizon et al. [37] supported this explanation by highlighting the effect of thickness on the translucency of different types of ceramics, with increased TP corresponding to decreased thickness. It was further clarified by Alayad et al. [18], finding that thinner material with a thickness of 0.5 mm showed significantly higher translucency than 1 mm. Also, Babaier et al. [38] findings were consistent, showing significantly higher TP values for 0.5 mm-thick than 1 mm-thick. Similar results were reported by Günal Abduljalil et al. [24]. Meanwhile, Gunal and Ulusoy [19] found significant differences in TP values for the two thickness of Vita Enamic even with the same shade and translucency level, which coincide with our results.

In the present study, the mean TP of Grandio was significantly higher than that of Vita Enamic for each thickness separately. These findings were in accordance with Babaier et al. [38], despite using high-translucency materials. Meanwhile, the Günal Abduljalil et al. [24] results were convenient; they measured the relative translucency parameter for both materials for each thickness (0.5 and 1 mm-thick). They used the same shade and translucency of the materials as in the current study. As mentioned above in the current study, the results of Vita Enamic demonstrated less translucency than Grandio at both thicknesses, attributing this to the structure of both materials. Vita Enamic has a double-penetrating polymer-infiltrated ceramic network [39]. It contains a significant amount of aluminium oxide (Al_2_O_3_) in its ceramic-matrix composition, enhancing opacity values. Moreover, this material is composed of metal oxide opacifiers like titanium oxide (TiO_2_) and zirconium oxide (ZrO_2_), which negatively affect light transmission [24, 31, 33]. Furthermore, the mismatch in refractive index between the polymeric matrix and ceramic phase causes increased opacity values due to multiple reflection and refraction at the matrix phase interface [33, 40]. The refractive indices of the UDMA, TEGDMA, TiO_2_, Al_2_O_3_ and ZrO_2_ are quoted as 1.48, 1.46, 2.49, 1.77 and 2.22, respectively [3, 24, 33]. As TiO_2_ has the highest refractive index among all, it exhibits the extreme mismatch with the resin-matrix, resulting in higher opacity in Vita Enamic [14]. While, Grandio is a nanoceramic with dispersed fillers like silica and barium glass embedded in a polymeric matrix [31, 41–43]. The refractive index of UDMA is close to those of silica (1.47) [44] and barium (~1.55) [14, 24] glass fillers. These nanoscale filler particles with diameters smaller than visible light wavelength reduce light scattering and enhance light transmission. This leads to enhanced translucency, thus explaining the higher TP value of the Grandio material containing nano-filler particles [3, 14, 24].

The TP values of veneer materials should be close to that of natural enamel to achieve a natural-looking aesthetic restoration [27]. The mean TP values of 1 mm-thick Vita Enamic in the current study (18.42) is similar to a previous study [45] which revealed a mean TP of 18.7 for a 1 mm section of natural human tooth enamel.

In the clinical situation, the degree and area of discolouration in one tooth are varied. The higher the ΔE_00_ value, the bigger and more noticeable the colour difference is to the human eye. The ΔE_00_ values in the current study revealed a significant decrease with an increase in material thickness for each tested material. Lambert’s law states that decreasing material thickness leads to decreased absorption, enabling more light transmission, while increasing thickness has inverse effects [13, 39]. Thin restorations enhance light transmittance, enhancing the colour properties of underlying tissues and resulting in a dramatic effect on the resulting colour [13]. Meanwhile, the increased thickness of the restoration enhances its opacity by reducing the reflective effects of the underlying substrate, effectively masking the dark base [29]. This rule explains the increase in masking efficiency and decrease in the calculated ΔE_00_ value due to thickness increases. Ongun et al. [13] investigated the influence of material thickness, cement shade and restorative material type on the final restoration colour of monolithic CAD/CAM blocks. The researchers detected lower ∆E_00_ values as a result of the increase in thickness on using blocks with a low translucency value, as in the current study. Contrarily to our findings, Günal-Abduljalil and Ulusoy [46] reported no significant difference in ΔE_00_ values between 0.5 and 1 mm thicknesses of Vita Enamic. This difference can be due to the fact that they used translucent cement under the samples.

Furthermore, in the current study, the highest masking ability was achieved by 1 mm Vita Enamic, followed by 1 mm Grandio, then 0.5 mm Grandio and 0.5 mm Vita Enamic. There were significantly lower ΔE_00_ values for Vita Enamic than Grandio at 1 mm thickness and inversely insignificant at 0.5 mm. These findings can be attributed to the differences in chemical compositions of the aforementioned tested materials that can cause colour coordinates alterations. Moreover, in the current study, the parametric factor K_L_ was set to 2 according to the CIEDE2000 formula (2:1:1) [20–23]. Nevertheless, previous studies have shown that texture affects light tolerance [20, 47, 48]; such assumption was supported by the results of Ongun et al. [13], who found that the L* value was greatly affected among other colour coordinates.

The null hypothesis of this study was partially rejected because there were significant differences in translucency and masking abilities among tested materials with different thicknesses, except for masking ability of 0.5 mm-thick of both materials showing no significant difference.

The required acceptability threshold is 1.78, as stated by Paravina et al. [22, 23] and Perez et al. [20]. From our results, Vita Enamic and Grandio demonstrated clinically acceptable colour difference values (1.22 and 1.66, respectively) at 1 mm thickness. At 0.5 mm thickness, the values (3.39 and 3.26, respectively) were above the clinical acceptability threshold.

In our study, we have used a PMMA tooth-coloured replica and a dark-stained one rather than natural teeth, which may be considered a limitation. However, this was done for better standardisation and the exclusion of other variables, as natural teeth may have diverse optical properties. Another limitation of the current study may be the use of a hand-held spectrophotometer. Even though bench spectrophotometers are suitable for in vitro studies, the VITA Easyshade V was chosen to precisely simulate the clinical situation. Furthermore, this clinical spectrophotometer provides accuracy and reliability, as reported by Dozić et al. [49] and Klotz et al. [50] for in vivo and in vitro research.

## Conclusions

Within the limitations of the current study, it can be concluded that:

1. The thickness of the veneering layer of the hybrid CAD/CAM materials is more effective for colour masking than their translucency.
2. In thin thickness, the masking ability is less effective, irrespective of the type of hybrid CAD/CAM materials.
3. The translucency of the hybrid CAD/CAM materials was affected by their composition.
4. Both hybrid CAD/CAM materials used in the study are promising alternatives for masking dark discolouration at 1 mm-thick.

## Data Availability

All data included in this study are available from the corresponding author upon request.
